# Gastrointestinal tract modeling using organoids engineered with cellular and microbiota niches

**DOI:** 10.1038/s12276-020-0386-0

**Published:** 2020-02-26

**Authors:** Sungjin Min, Suran Kim, Seung-Woo Cho

**Affiliations:** Department of Biotechnology, Yonsei University, Seoul, 03722 Republic of Korea

**Keywords:** Stem-cell biotechnology, Stem-cell niche

## Abstract

The recent emergence of organoid technology has attracted great attention in gastroenterology because the gastrointestinal (GI) tract can be recapitulated in vitro using organoids, enabling disease modeling and mechanistic studies. However, to more precisely emulate the GI microenvironment in vivo, several neighboring cell types and types of microbiota need to be integrated into GI organoids. This article reviews the recent progress made in elucidating the crosstalk between GI organoids and components of their microenvironment. We outline the effects of stromal cells (such as fibroblasts, neural cells, immune cells, and vascular cells) on the gastric and intestinal epithelia of organoids. Because of the important roles that microbiota play in the physiology and function of the GI tract, we also highlight interactions between organoids and commensal, symbiotic, and pathogenic microorganisms and viruses. GI organoid models that contain niche components will provide new insight into gastroenterological pathophysiology and disease mechanisms.

## Tissue engineering: realistic gastrointestinal tract models

Miniature guts and stomachs grown in the laboratory together with supporting cells and microbes provide a physiologically relevant model for studying gastrointestinal disease and testing candidate drug therapies. In a review article, Seung-Woo Cho and colleagues from Yonsei University, Seoul, South Korea, discuss the latest approaches scientists and drug developers are using to coax collections of stem cells into gut-like structures that best reproduce the biology of the human stomach and intestines. To precisely emulate the gastrointestinal environment, researchers have begun integrating cells from neighboring connective tissues, including structural cells, neural cells, and immune cells, along with resident bacteria and viruses that contribute to health and disease. Techniques such as bioprinting and microfluidics, and new biomaterials are being used to mimic the three-dimensional architecture of the digestive tract on a small scale.

## Introduction

The organoid is a miniaturized organ generated from stem cells grown in a dish. Various kinds of organoids can be produced depending on the target organs, such as the brain, liver, lung, kidney, stomach, and intestine^1–6^. Organoids contain various organ-specific cell types within a three-dimensional (3D) structure, thus containing the specific phenotype and physiology that each organ possesses. Organoids with these properties better mimic the in vivo environment than two-dimensional (2D) cell models, so they can be used for disease modeling and drug screening. In comparison with traditional animal models, organoids are cheaper and faster and are considered more ethical; further, their derivation from human stem cells enables better modeling of human physiology^7^. Moreover, organoids can be easily genetically manipulated, permitting the study of organogenesis and disease development^8^.

Despite the many advantages of organoids, there remain some limitations arising from scalability and reproducibility. Another major problem involves differences between organoids and actual organs due to the absence of surrounding tissue, such as the stromal cells that are present in connective tissue of any organ. Although the types of stromal cells are slightly different for each organ, they generally include fibroblasts, neural cells, immune cells and vascular cells. They are known to have a profound effect on an organ through immune responses, nutrient supply, paracrine signaling and extracellular matrix supply^9–12^. Thus, including these cell types during organoid culture is important for increasing the maturity and complexity of organoids^13^.

Organoid models of the gastrointestinal (GI) tract, intestinal and gastric organoids, were first developed in 2009 and 2010, respectively^14,15^. Researchers extracted Lgr5^+^ adult stem cells (ASCs) directly from the stomach and intestine and cultured them with the appropriate growth factors and supporting matrix. As described above, both of the resulting organoids consisted only of organ-specific tissue (gastric or intestinal epithelium), while other tissue types were excluded. ASC-derived GI organoids consist only of gastric epithelial cells such as parietal cells, chief cells, and surface pit cells or intestinal epithelial cells such as enterocytes, Paneth cells, and goblet cells. On the other hand, the generation of intestinal and gastric organoids from pluripotent stem cells (PSCs) was reported in 2011 and 2014, respectively^5,6^. Since PSCs themselves have the capacity to differentiate into three germ layers (ectoderm, mesoderm, and endoderm), mesenchymal cells, including smooth muscle cells, fibroblasts, and myofibroblasts, are co-developed with PSC-derived GI organoids during differentiation. To date, the presence or absence of mesenchymal lineage cells has been known to be a main difference in the cell components of PSC-derived and ASC-derived organoids^16,17^. The presence of a small number of mesenchymal cells enables epithelial-mesenchymal interactions and eventually allows the successful differentiation of GI organoids^18^. However, the co-development of organoids with other environmental cells, such as neural cells, immune cells, and endothelial cells, has rarely been reported. This results in differences in the environment between the native organ and the organoid, which prevents the organoid from properly recapitulating the actual organ. To address this problem, many researchers are attempting to improve organoid culture using a variety of methods, such as three-dimensional (3D) bioprinting, biomaterials, and coculture^19,20^. In particular, GI organoids are closely related to the microbiota. The organs of the GI tract have a mucosal surface on the lumen side, allowing various microbes to colonize there^21^. The microbes reside in the gut and influence intestinal biology, such as epithelial turnover, physiological processes and immune homeostasis, and they compete with other pathogens that enter through the diet and have a nonnegligible effect on the host^22,23^. In fact, the intestinal microbiota affects drug pharmacokinetics and treatment outcomes, and its importance is becoming increasingly evident in drug discovery and clinical trials^23,24^. To increase the understanding of drug mechanisms, it seems necessary to incorporate microbiota into GI organoids. In addition to symbiosis with the host, some microbiota have a tremendous effect on severe GI tract disease. For example, *Helicobacter pylori* is the main cause of stomach diseases such as peptic ulcers, chronic gastritis, and gastric cancer, and intestinal microbes and viruses lead to diarrhea, inflammation, colitis, inflammatory bowel disease and even obesity^25–27^. The coculture system of microbiota and GI organoids would facilitate the study of GI tract diseases in terms of host-pathogen interactions^28^. Furthermore, due to the unique culture environment of specific microorganisms and viruses, organoids can be used as a platform to grow such species that previously have been difficult to maintain.

**Fig. 1:**
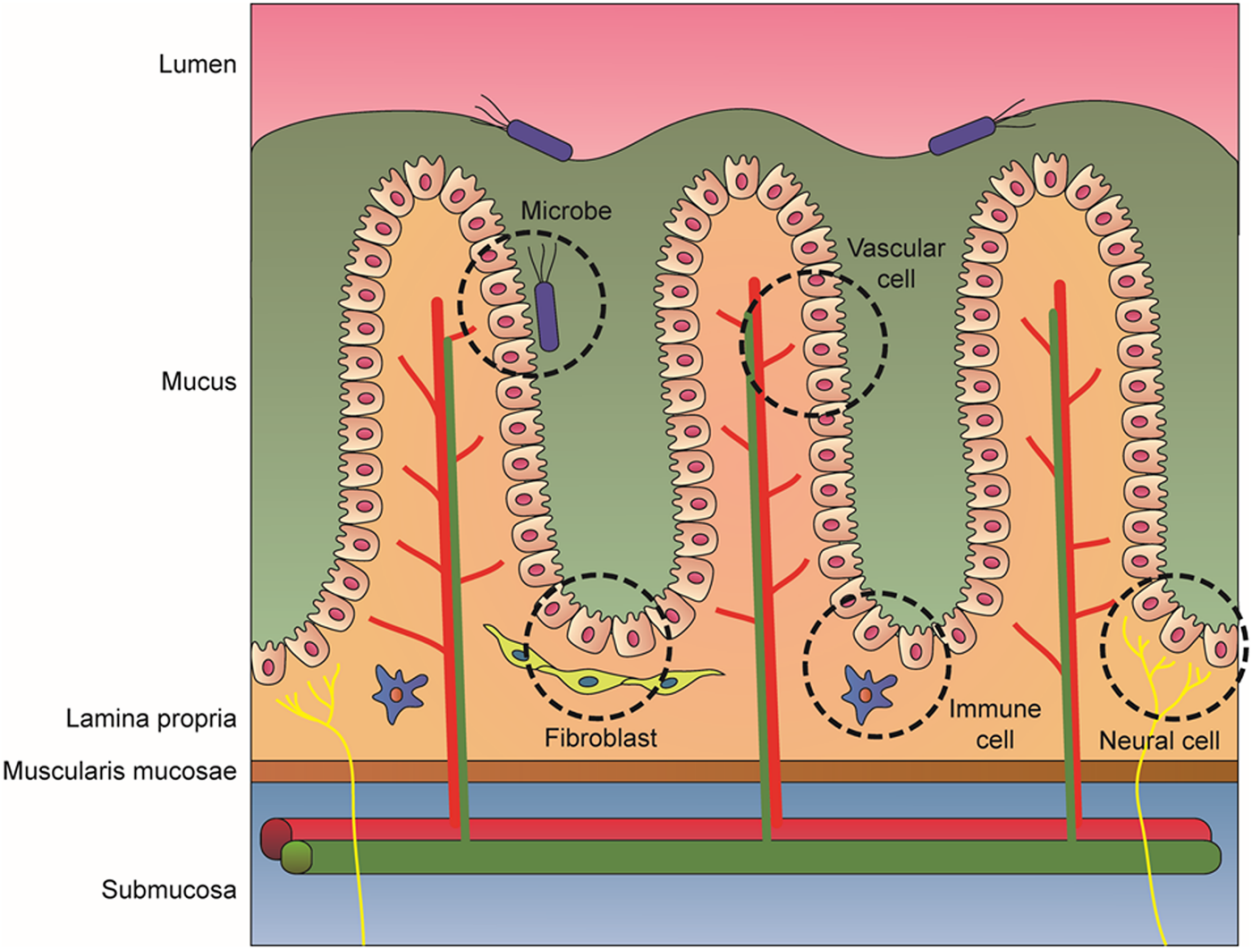
Image of GI epithelium and the surrounding tissues. In GI tissues, there are numerous interactions between the epithelium and microbes or other cells, such as fibroblasts, vascular cells, immune cells and neural cells. Their communication has significant effects on the functions and homeostasis of the epithelium, indicating the necessity of including stromal cells and microbiota for producing functional 3D organoid models of the GI tract.

**Fig. 2:**
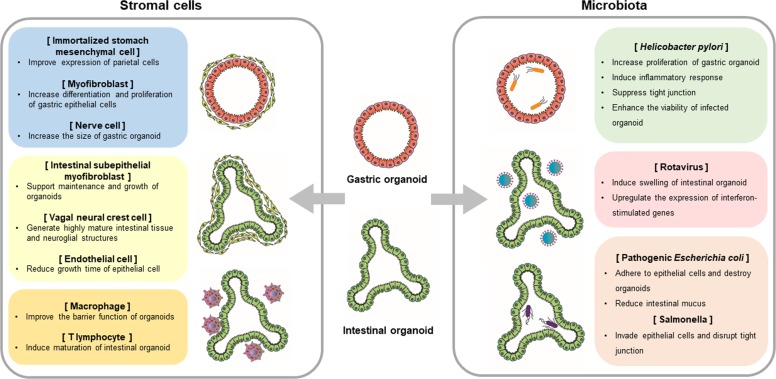
Coculture models of GI organoids with stromal cells and microbiota. The biological effects of various stromal cells and microbiota on GI organoids in coculture models are described.

In this review, we introduce GI organoids integrated with cellular and microbiota niche components as tools for modeling the physiology and pathology of the GI tract (Fig. 1). First, we describe platforms involving the coculture of GI organoids with various stromal cells that reside in the native organ (Fig. 2). Then, we discuss how inoculation of GI organoids with microbiota can be used to investigate the pathophysiological effects of microorganisms and viruses on the GI tract (Fig. 2). These engineered GI organoids provide an effective alternative to conventional cell-based in vitro models and animal models for drug development and for studying GI diseases.

## Gastric organoids integrated with stromal cells

The stomach is a complex organ containing various cell populations. It is comprised of the mucosa, submucosa, muscularis propria and serosa, which are organized into four closely connected layers. The gastric epithelium is divided into two main parts, the corpus (fundus) and antrum, which each contains different cell types. The epithelium of the corpus region contains many functional cells, including acid-secreting parietal cells and pepsinogen-secreting chief cells, while the epithelium of the antrum region consists mostly of mucus-producing cells. The lamina propria is a loose connective tissue under the gastric epithelium and contains many surrounding stromal cells, such as fibroblasts and immune cells. Nerves and blood vessels populate the submucosal region of the stomach, where they are thought to interact very closely with the gastric epithelium based upon their histological proximity^29^. However, the precise cellular interactions and their effects on the gastric epithelium remain unclear.

As mentioned, surrounding mesenchymal cells provide the gastric epithelium with a specific cellular niche in the stomach, but these cells do not exist in ASC-derived gastric organoids. To incorporate epithelial-mesenchymal interactions in organoids, immortalized stomach mesenchymal cells (ISMCs) were cocultured with gastric organoids^30^. Coculture of organoids with ISMCs increased the number of all cellular components of the fundus (e.g., parietal cells, chief cells, surface pit cells, and mucous neck cells), which is a part of the stomach, and there was improved functionality of parietal cells^30,31^. Moreover, an integrated system comprised of glandular stomach cells and gastric mesenchymal myofibroblasts increased the differentiation and proliferation of gastric epithelium and supported their long-term maintenance^32^. These findings suggest that mesenchymal cells are important not only for gastric patterning during embryonic development but also for maintenance and functional activity of the gastric epithelium^33,34^. Wnt signaling is also known to be a crucial factor for the proliferation of gastric stem cells, and R-spondin (Rspo), which is provided by the surrounding stromal cells, enhances Wnt signaling. Consistent with this, organoid growth was shown to be supported in Rspo-free medium as long as the gastric organoids were cocultured with stromal cells, because endogenous Rspo was produced by stromal myofibroblasts^35^. This suggests that stromal myofibroblasts have a significant effect on the turnover and regeneration of the gastric epithelium.

The enteric nervous system (ENS), which interacts closely with the gastric epithelium, extends through the GI tract from the esophagus to the anus. The ENS is called a “second brain” because it interacts with the central nervous system (CNS) in both directions and can function without instructions from the brain and CNS. This autonomous system has significant effects on the motility, absorption of nutrients and hormone secretion of the digestive system^36,37^. Within the stomach, neurons of the ENS are primarily involved in gastric acid secretion and the release of histamine and 5-hydroxytryptamine^38^. Coculture of gastric organoids with nerve cells derived from the myenteric plexus was found to enhance organoid growth, and this effect was attributed to Wnt signaling^39^. However, further studies utilizing such coculture models are required to understand the mechanism of interaction between the ENS and the gastric epithelium.

The stomach is protected from external infections and diseases by the barrier function of the gastric epithelium combined with an immense contribution of immune cells. The interaction between immune cells and the epithelium is closely linked to the gastric pathophysiology of bacterial infection, gastric cancer, and gastric repair^40–42^. Chakrabarti et al. established a model system for predicting the effect of immune checkpoint inhibitor therapy using a coculture system of gastric cancer organoids and immune cells^43^. Expression of programmed death 1 (PD-1) in cytotoxic T lymphocytes (CTLs) was induced by gastric cancer organoids in the presence of dendritic cells (DCs), and the interaction of PD-1 with programmed death ligand 1 (PD-L1) in gastric cancer cells inhibited tumor destruction. In organoid-based systems that provide coculture with immune cells or conditioned media of immune cells, pharmacological inhibition of the PD-L1 interaction with PD-1 was found to increase apoptosis of tumor cells. Although more research is required, gastric organoid models incorporating immune cells show promise as in vitro preclinical tools suitable for patient-specific immunotherapy.

## Gastric organoids infected with *Helicobacter pylori*

*H. pylori* is the leading cause of fatal gastric diseases, including gastric cancer and peptic ulcer disease^27,44^. To study the pathogenesis of *H. pylori* and validate experimental drugs, an effective model system that can recapitulate the pathophysiology of the human stomach is needed. The 3D epithelial architecture of gastric organoids provides an in vitro model for infection studies that is superior to 2D cell cultures^45^, and they are more cost-effective and time-effective than animal models. Infection studies utilizing gastric organoids have revealed numerous mechanistic insights. Upon microinjection of *H. pylori* into PSC-derived gastric organoids, the CagA protein of *H. pylori* was shown to bind to the c-Met receptor of organoid epithelial cells, leading to phosphorylation of c-Met and consequent activation of epithelial cell proliferation^5^. Although the effects of *H. pylori* on gastric epithelial cells, including increased proliferation and cell motility, have been reported in 2D conditions^46,47^, conventional 2D models lack diversity and polarity of epithelial cells, so they cannot accurately recapitulate the interactions between bacteria and host cells^48^. In contrast, microinjection of *H. pylori* into the cavity of 3D organoids with polarized epithelial structures can emulate physiological changes and pathological events following *H. pylori* infection in the stomach better than 2D epithelial cell models with *H. pylori* simply added to the culture medium. Microinjection of *H. pylori* into ASC-derived human gastric organoids also confirmed that *H. pylori* activates the NF-κB pathway and chemokine IL-8, which is a target of NF-κB, resulting in an inflammatory response^49^. Boccellato et al. successfully generated a polarized gastric epithelial layer from a gastric organoid using an air-liquid interface and confirmed that all phenotypes seen in the antral region of the stomach, including mucus production, are observed in this layer^50^. Investigating the distinct responses of the gastric epithelium to *H. pylori* using this model system, it was also found that undifferentiated basal cells had a stronger inflammatory response than foveolar cells.

The organoid model system can be used to validate established hypotheses and discover new mechanisms of *H. pylori* pathology. For example, it has been known that *H. pylori* promotes proliferation of the gastric epithelium, but the detailed mechanism behind this occurrence is unclear. Using gastric organoids, Bertaux-Skeirik et al. showed that CD44, which was already known to be associated with c-Met, has significant involvement in the epithelial proliferation caused by CagA-positive *H. pylori*^48^. They also confirmed that atrophic gastritis caused by *H. pylori* was repressed by treatment with a CD44 peptide inhibitor. Another study found that the proliferation of gastric organoids infected with *H. pylori* is regulated by CagA and β-catenin signaling, and mislocalization and suppression of the cancer-associated tight junction protein claudin-7 is induced by β-catenin and snail activation^51^; these data increase our understanding of the mechanism by which *H. pylori* causes gastric cancer. Huang et al. also used gastric organoids to study how *H. pylori* is attracted to and colonizes the gastric epithelium^52^. They revealed that the *H. pylori* chemoreceptor TlpB recognizes urea, a metabolite produced by gastric organoids, causing *H. pylori* to be rapidly attracted to the epithelium, where it can modify the gastric microenvironment by hydrolyzing urea with urease.

To account for the important role of the immune response in disease, immune cells have been introduced into organoids. *H. pylori* infection was shown to increase the expression of PD-L1 in gastric epithelial cells via the Sonic hedgehog (Shh) signaling pathway^53^. Based on the fact that PD-L1 expression is associated with the immune system^54^, DCs and CTLs were cocultured with gastric organoids to study the interaction between PD-L1 and PD-1 in an infected gastric epithelium^53^. The increased expression of PD-L1 in *H. pylori*-infected organoids increased organoid viability through the interaction of PD-L1 with PD-1 that was expressed by CTLs, while pharmacological inhibition of PD-1 blocked this interaction, inducing death of infected epithelial cells. In a mouse model of infection, DCs were recruited to the gastric epithelium, where they interacted directly with *H. pylori*^55^. Similar findings were recapitulated using a gastric organoid model, where monocyte-derived DCs were found to migrate to and interact with uninfected gastric epithelial organoids, and their migration was increased during *H. pylori* infection^56^. This augmented recruitment of DCs was shown to be caused by multiple chemokines derived from infected gastric organoids, while the recruited DCs played a role in the phagocytosis of *H. pylori*, similar to what occurs in gastric mucosa in vivo. As such, gastric organoids have proven to be an excellent in vitro infection model that can overcome the limitations of existing model systems, and they are expected to accelerate the development of etiological research.

## Intestinal organoids integrated with stromal cells

The intestinal epithelium is the innermost part of the mucosa layer surrounding the intestinal lumen, where direct encounters with nutrients and foreign substances entering the GI tract occur. It is composed of specific intestinal cells that play important roles in digestive, absorptive, protective and secretory functions. The intestinal lamina propria, located beneath the epithelium, comprises various types of mesenchymal cells, such as fibroblasts, myofibroblasts, mural cells, immune cells, and smooth muscle cells of the muscularis mucosae^57,58^. Dense networks of blood vessels and nerves extend throughout the intestinal layers, assisting with the diverse functions of the intestine^59–61^. However, ASC-derived intestinal organoids, which recapitulate only the intestinal epithelium, and PSC-derived intestinal organoids, which mimic intestinal epithelium and some mesenchymal cells, lack these complex intercellular interactions. Thus, similar to gastric organoid models, the coculture of intestinal organoids with multiple cell types has been implemented to better simulate the in vivo intestinal cellular niche^19,62^.

Intestinal subepithelial myofibroblasts (ISEMFs) provide pivotal cues regarding the intestinal stem cell niche by secreting growth factors such as Wnt ligands and BMP antagonists^57,63,64^. Coculture of ASC-derived intestinal organoids with ISEMFs permitted long-term culture of organoids in the absence of some growth factors necessary for organoid culture^65^. The presence of ISEMFs also promoted the formation of larger and more complex intestinal organoids than what was observed in monocultured organoids, and the conditioned medium of ISEMFs also promoted intestinal organoid growth^66^. These organoids cocultured with ISEMFs were exhibited more successful engraftment and proliferation upon transplantation. Coculture with ISEMFs also aided the expansion of mouse intestinal organoids, even in collagen gels^67^. In the case of human intestinal organoids, monocultured organoids seeded in collagen gels underwent autolysis within 2–3 days; however, coculture with ISEMFs or supplementation with the conditioned medium from ISEMFs enabled rapid growth while maintaining a 3D structure in the collagen gel^68^. In an organotypic cell culture system consisting of an additional acellular layer and a layer with cells on a Transwell^®^ permeable support system, intestinal organoids cocultured with intestinal myofibroblasts also showed increases in diameters and improved viability^39^. These results indicate that ISEMFs provide useful niche factors for intestinal stem cells and for the growth of intestinal organoids.

The ENS is an indispensable element involved in the manipulation of various intestinal functions, such as peristalsis, coordination of blood flow, regulation of the intestinal epithelial barrier and secretion of fluid and electrolytes^36,69,70^. However, both ASC-derived intestinal organoids and PSC-derived intestinal organoids lack ENS direction. To address this shortcoming, efforts have been undertaken to differentiate PSCs into vagal neural crest cells (NCCs), from which the ENS is mainly derived, and integrate them into organoids^71,72^. Fattahi et al. established a method for neural differentiation and maturation of enteric neural crest (ENC) precursors and confirmed the in vivo interactions of the precursor cells with mouse primary intestinal tissue using a tissue engineering approach^71^. Organoid units extracted from mouse intestines were directly and simultaneously seeded with human PSC-derived ENCs on a polyglycolic acid scaffold, and after implantation, they formed an intestine-like structure that expressed human neural cell markers in both the epithelial and muscle layers. Workman et al. generated intestinal organoids with functional neurons and glia by mechanical aggregation of PSC-derived mid/hind spheroids with PSC-derived NCCs; following transplantation into mice, these organoids developed into highly mature intestinal tissue with neuroglial structures similar to those of a myenteric and submucosal plexus^72^. In addition, organoids integrated with NCCs derived from PSCs with PHOX2B mutations, which are associated with complete aganglionosis of the bowel, grew poorly and showed a pattern of suppressed smooth muscle development. These findings indicate that NCCs have noncellular autonomous effects on the development of intestinal cells and that these engineered organoids can be used as a specific in vitro model of Hirschsprung’s disease. It has also been shown that PSC-derived intestinal organoids integrated with neural and muscle cells can be generated by differentiating the PSCs into the three germ layers using a strategy that mimics in vivo organogenesis^73^. These integrated intestinal organoids expressed the enteric neural marker known as protein gene product 9.5 (PGP9.5), had neurofilaments distributed throughout the mesenchymal area and exhibited contractile movement that could be controlled by treatment with histamine and atropine.

The ability to mimic the intestinal immune system provides another important objective for organoid models. Since the intestinal lumen is continuously in contact with foreign materials and enteric commensal microbes, a defense system is essential for protecting the body from invasion. The intestine has special structures called gut-associated lymphoid tissues (GALTs) that contain specialized M cells, which serve as an inductive site for the mucosal immune response; GALTs also contain many different immune cells, such as macrophages, DCs, T cells, B cells, plasma cells and mast cells, which play a key role in immune homeostasis^74–77^. To facilitate the interaction between intestinal epithelial cells and immune cells, Noel et al. seeded intestinal stem cell-derived organoids on a Transwell^®^ permeable support to generate epithelial monolayers that were cocultured with monocyte-derived macrophages^78^. The presence of macrophages enhanced the height and transepithelial electrical resistance of epithelial cells, suggesting that they have positive effects on the maturation and barrier functions of the intestinal epithelium. Upon infection with pathogenic *Escherichia coli* (*E. coli*) to mimic in vivo luminal exposure, the cocultured macrophages sensed *E. coli* and extended toward the epithelium. In addition, the number of viable *E. coli* was reduced, and the breakdown of the epithelial barrier was partially protected compared with that of the epithelial monoculture model. This coculture model provides insight into host-pathogen interactions and the innate immune response in the intestinal mucosa.

Unlike the above method of interrogating the direct effects of immune cells on intestinal organoids, some studies have investigated the indirect effects of immune cells on intestinal organoids using interleukins. ASC-derived intestinal organoids that were cocultured with lymphoid cells, which produce interleukin-22 (IL-22), were significantly larger than monocultured organoids^79^. Similarly, when recombinant IL-22 was added to the culture medium, an improvement in intestinal organoid growth was also found. This indicated that the immune system is a component of the intestinal stem cell niche and regulates intestinal regeneration. In a PSC-derived intestinal organoid system, coculture with T lymphocytes and treatment with interleukin-2 (IL-2) led to in vitro maturation of intestinal organoids that retained fetal intestinal properties^80^. Jung et al. confirmed that IL-2, the most abundant cytokine secreted by T lymphocytes, produces PSC-derived intestinal organoids with a gene expression profile and functionality that is similar to that of adult intestinal tissue^80^. These results indicate that soluble factors secreted by immune cells have a profound effect on the regeneration and maturation of intestinal tissue and that inclusion of immune cell interactions is an effective strategy for overcoming the limitations of functionally immature PSC-derived intestinal organoids.

Although blood vessels are crucial for intestinal function, the introduction of vasculature into intestinal organoids has rarely been attempted. Kasendra et al. used microfluidic devices comprised of two parallel microchannels separated by a porous polydimethylsiloxane (PDMS) membrane to combine the intestinal epithelium and the microvascular endothelium in vitro^81^. To generate a vascularized intestine-on-a-chip, intestinal epithelial cells dissociated from organoids were seeded on the top of the PDMS membrane, and intestinal microvascular endothelial cells were plated underneath the PDMS membrane. The presence of endothelial cells helped to shorten the growth time of the intestinal epithelial cells, and this vascularized intestine unit in the chip also exhibited genetic profiles similar to those of the in vivo duodenum. While they used microfluidic devices to attract vessel networks to the intestinal organoid, other researchers used in vivo transplantation to induce spontaneous vascularization. Watson et al. implanted intestinal organoids differentiated from human PSCs into a mouse kidney, which is a highly vascularized tissue^82^. These transplanted organoids not only became more mature but also had blood vessels that extended from the host tissue.

## Intestinal organoids that incorporate microbiota and viruses

The intestine is a complex ecosystem composed of myriad coexisting bacteria, fungi and viruses that influence GI physiology^83,84^. The intestinal microbiota defends against the growth of invading bacteria and breaks down food to assist nutrient absorption by intestinal epithelial cells. They also produce metabolites to regulate immunity and other physiological activities. Therefore, when the balance of the intestinal ecosystem is perturbed, various diseases, such as inflammatory bowel disease and obesity, can occur^85^. Because the intestinal microbiota plays a role as a third organ^86^, interest in it among the research community, as well as the food industry is rapidly increasing. To meet these demands, several studies of intestinal microbial interactions have been conducted in an in vitro environment using intestinal organoids.

Some researchers have studied the effects of coculture with intestinal organoids and commensal bacteria such as *Lactobacillus*, a probiotic species that enhances the barrier function of the intestinal epithelium. Coculture with *Lactobacillus rhamnosus* increased organoid proliferation and differentiation into Paneth cells^87^, while *Lactobacillus reuteri* D8 improved the growth of intestinal organoids and protected against damage caused by the proinflammatory cytokine TNF-α^88^. In addition, Hill et al. microinjected the nonpathogenic *Escherichia coli* strain ECOR2 into the lumen of PSC-derived intestinal organoids to establish an in vitro model for exploring host-bacterial symbiotic interactions in the neonatal intestine^89^. After microinjection, ECOR2 grew rapidly inside the lumen, and the organoid was maintained and did not become disrupted, confirming that both organisms were being stably cocultured. Interestingly, the stimuli by bacterial contact increased barrier function and antimicrobial defense and enhanced tissue maturation. These organoid-based microbial studies provided a foundation for revealing the mechanism by which probiotics contribute to human health.

In addition to commensal bacteria, pathogenic bacteria *Escherichia coli*^90–93^, *Salmonella*^94–96^, *Clostridium difficile*^97^, and *Cryptosporidium*^98^ can be incorporated into intestinal organoid systems to understand the effects of bacterial pathogens on the intestinal epithelium. Colonization of enterohemorrhagic *E. coli* (EHEC) in a human colonic organoid monolayer decreased intestinal mucus and destroyed microvilli, facilitating bacterial access to and infection of epithelial cells^93^. The mechanism was found to involve a reduction of brush border resident protein protocadherin 24 (PCDH24) by the EHEC protease EspP, demonstrating the ability of the model system to uncover the molecular mechanisms responsible for early intestinal colonization. The addition of *Salmonella enterica serovar Typhimurium* to organoid culture medium diminished intestinal epithelial tight junctions and led to bacterial invasion, with infected organoids exhibiting pathophysiological features such as NF-κB pathway activation and stem cell reduction^94^. To establish an in vitro human model for *Clostridium difficile* infection or *Cryptosporidium parvum* infection, direct contact between the lumen of intestinal organoids and bacteria was achieved using a microinjection technique^97,98^. *Clostridium difficile* persisted in the organoids and produced toxins TcdA and TcdB, which have the ability to destroy the intestinal epithelium^97^. *Cryptosporidium parvum* propagated inside the organoid and completed its entire life cycle, which is only possible inside a suitable host^98^.

Enteric viruses are also major disease agents that easily enter the intestine through water or food. Their mechanisms of propagation and replication were impossible to study in vitro prior to the development of organoids. Rotavirus and norovirus are the most common viruses that cause GI disease. Finkbeiner et al. observed that intestinal organoids supported the replication of rotavirus isolated directly from stool samples, despite this virus being previously difficult or impossible to culture^99^. Moreover, rotavirus was found to infect not only intestinal epithelial cells but also adjacent mesenchymal cells^99^, providing a new perspective on rotavirus pathophysiology. Rotavirus could also replicate in organoids derived from three different regions of the small intestine, all of which recapitulated in vivo characteristics, such as host susceptibility and preferential infection of differentiated cells^100^. Organoids have been used to construct personalized infection models with rotavirus derived from individual patients as a way to evaluate patient-specific antiviral therapy^101^. Although human norovirus (HNV) has been difficult to culture in transformed epithelial cell lines and animal models, an intestinal organoid-derived monolayer supported a productive and complete HNV replication cycle, and diverse HNV strains were able to replicate in this system^102^.

Intestinal organoids offer a superior in vitro model system for the cultivation of microbiota that influence GI physiology and for understanding how they encounter the intestinal epithelium and cause disease. Mechanistic details obtained from such modeling may provide new avenues for the prevention and treatment of many GI tract diseases.

## Conclusions and outlook

Organoid models are increasingly being used to explore biological phenomena associated with tissue development and differentiation, as well as human diseases. GI organoid-based integrated systems provide in vitro models that more accurately mimic the in vivo environment of the GI tract than previous culture systems, and they lay the foundation for deeper insights into interactions of the GI epithelium with cellular/microbiota niches. Several studies have utilized engineered organoid systems to elucidate how surrounding cells or microbiota affect GI pathophysiology. However, current techniques with multiple cocultures and strategies for the reconstitution of culture environments need to be further improved to enhance the structural complexity and functional maturation of engineered organoids. Current methods of GI organoid coculture are mostly performed by simple mixing components, which fails to recapitulate the regularly arranged structures observed in vivo. Moreover, there is a need for the development of new culture systems that can individually supply different culture media that are suitable for different cell types. The focus must be not only on surrounding cells and microbiota but also on the surrounding extracellular matrix (ECM) because most research to date has utilized Matrigel^®^, which does not provide a tissue-specific environment. Various technologies are being developed for this purpose, such as 3D bioprinting and the use of microfluidic devices and biomaterials^103–108^. Bioprinting has the capacity to encapsulate various cells in a compatible hydrogel and to place them in the desired position suitable for coculture. This is an automated and efficient system that can achieve tissue complexity by incorporating a complex network of vasculature and innervation^103,109^. Microfluidic devices can be fabricated such that different cells can be seeded in different zones, making these devices ideal for coculture^110^. For example, angiogenesis can be mimicked by placing organoids and vascular cells in different locations and connecting them using soluble factors^104^. Microfluidic devices are also well suited for modeling because of their ability to provide fluid flow that is similar to what is observed in vivo. The coupling of a microfluidic device with an organoid has recently been termed “organoid-on-a-chip”, and this represents an innovative approach in biomedical research^105,106^. Moreover, various engineered materials for organoid culture containing a decellularized matrix and a chemically defined matrix are being actively developed^106,107,111^.

**Fig. 3:**
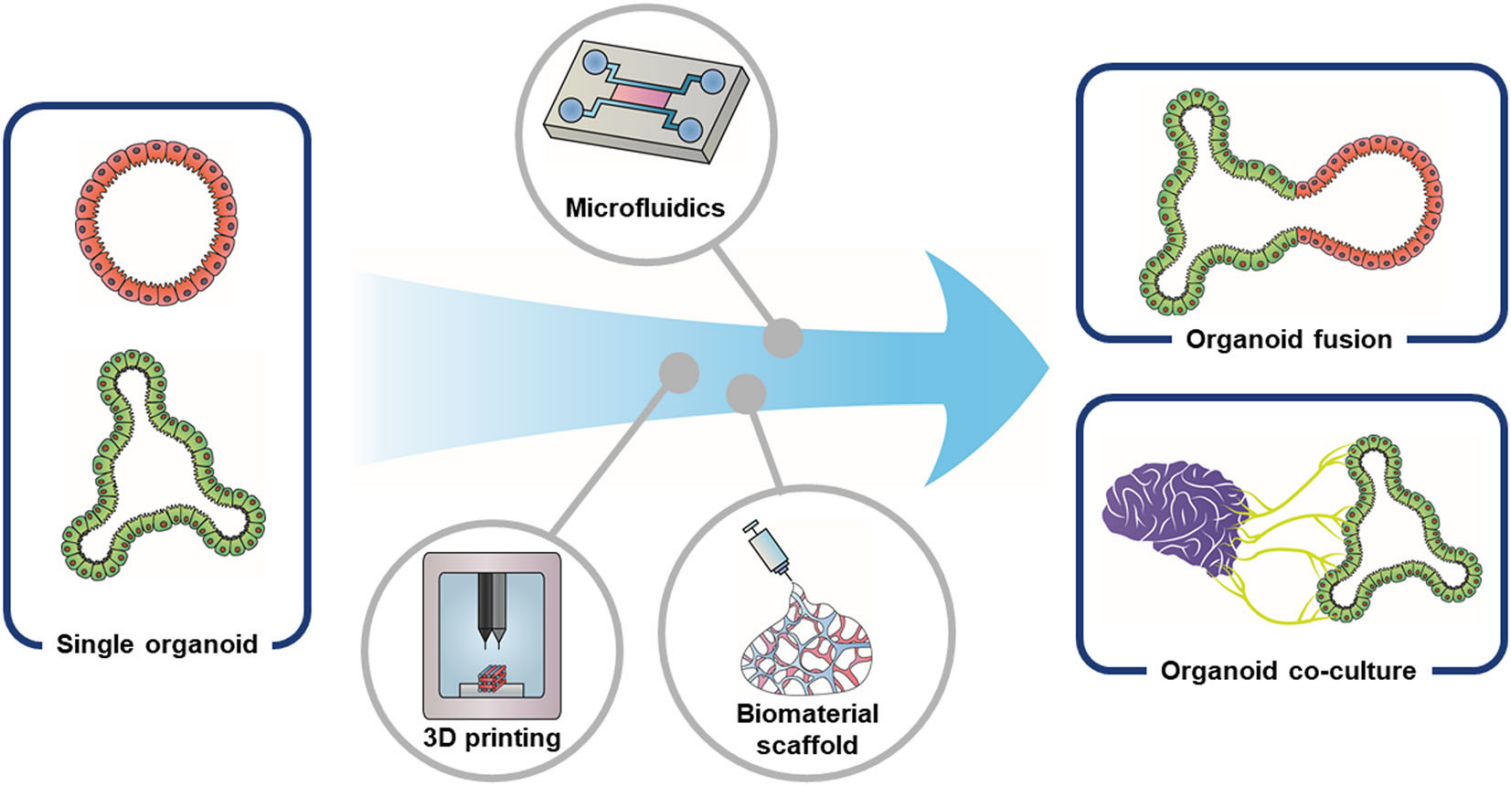
Bioengineered platforms for developing GI organoids assembled with other types of organoids. Through bioengineering techniques such as 3D bioprinting and the use of microfluidics and biomaterials, a novel integrated GI system can be developed by connecting the various organoids that make up the digestive system or by coculturing with completely different types of organoids such as brain organoids. These innovative organoid systems enable the modeling of highly complex human physiology, such as reciprocal regulation between the GI tract and other major organs, and pathological phenomena associated with diseases.

Methods employing the fusion of organoids and coculture of multiple organoids have recently emerged (Fig. 3)^112–114^. A multiorgan integrated system fusing three different types of digestive system organoids on a microfluidic device has been used to identify crosstalk between organoids^113^. A new protocol for generating integral multiorgan structures of the digestive system has also been reported, leading to the fusion of two PSC-derived spheroids that correspond to the anterior and posterior gut, as well as to the creation of hepato-biliary-pancreatic organoids that establish boundary interactions between two spheroids without any extrinsic factors^114^.

Drug screening with only one type of organoid can be a limiting factor because the in vivo degradation and absorption of a drug occurs through various digestive organs. In particular, the gut and liver communicate with each other through portal veins and biliary tracts, enabling microbial products such as microbial-associated molecular patterns (MAMPs) to travel from the gut to the liver, causing inflammation and liver damage^115^. Liver diseases such as nonalcoholic fatty liver disease (NAFLD), alcoholic liver disease (ALD), cirrhosis, and hepatocellular carcinoma (HCC) are also reported to be associated with changes in the gut microbiome. The development of an integrated system consisting of several digestive organoids and microbiota makes it possible to screen drugs under conditions that mimic an in vivo environment.

Gut-brain crosstalk is closely related to homeostasis of the GI tract and cognitive ability of the brain^116^. In particular, autism spectrum disorder (ASD), cerebral palsy (CP), and spina bifida, which are neurodevelopmental diseases, have been reported to be associated with GI abnormalities^117,118^. Therefore, a novel model system created by the coculture of GI organoids with brain organoids could be used to study the mechanisms underpinning these disorders. Likewise, the development of systems integrating various other organoids may elucidate many other physiological and pathological conditions throughout the body.
